# HSS@Home, Physical Therapist-Led Telehealth Care Navigation for Arthroplasty Patients: A Retrospective Case Series

**DOI:** 10.1007/s11420-019-09714-x

**Published:** 2019-08-22

**Authors:** Charles Fisher, Elizabeth Biehl, Matthew P. Titmuss, Rachelle Schwartz, Chandra Sekhar Gantha

**Affiliations:** Hospital for Special Surgery, 535 East 70th Street, New York, NY 10021 USA

**Keywords:** telehealth, arthroplasty, physical therapy

## Abstract

**Background:**

As the rate of total joint arthroplasties performed in the USA continues to increase, so does the push for more value-based care. Bundled payments have encouraged organizations to be creative in limiting care overuse. Telehealth is one option for caring for arthroplasty patients post-surgery while limiting costs and improving communication with the surgical team.

**Questions/Purposes:**

We sought to determine the effects of the implementation of HSS@Home, a telehealth rehabilitation program that uses patients’ existing technology, in patients after they had undergone total knee or total hip arthroplasty.

**Methods:**

In this retrospective case series, of 32 patients referred, 19 patients (nine men and ten women; average age, 69 years) were enrolled in HSS@Home after undergoing a pre- and post-operative screening process. Telehealth video visits were conducted, wherein a physical therapy navigator assisted patients in following exercise and mobility programs, addressing patients’ concerns while transitioning to outpatient therapy. Patients were seen within 24 h of hospital discharge, 3 times a week for 3 weeks, for an average of 11 sessions. Episodes of care were recorded in the patient’s electronic medical record.

**Results:**

There were no readmissions among the 19 patients. Nurse practitioners were consulted for all patients, predominantly for non-emergent reasons. Feedback from patients and physicians was positive, and no overutilization of care was found.

**Conclusion:**

HSS@Home was a promising alternative to live, in-home physical therapy that was effective in monitoring this series of patients after hip or knee arthroplasty. This preliminary data sets the stage for further research into the use of telehealth technology to provide rehabilitative care to arthroplasty patients.

**Electronic supplementary material:**

The online version of this article (10.1007/s11420-019-09714-x) contains supplementary material, which is available to authorized users.

## Introduction

The annual number of total joint arthroplasty (TJA) procedures performed in the USA is growing and expected to reach nearly 4 million by 2030, with many of these patients requiring rehabilitation services post-operatively [9]. Following TJA, a number of post-discharge options is available for patients to continue their recovery, including discharge to extended care facilities such as skilled nursing or acute rehabilitation facilities for patients with limiting comorbidities or requiring intensive rehabilitation, as well as discharge home with home-based physical therapy (PT) immediate outpatient PT, or no services. The annual cost of post-arthroplasty rehabilitation in the USA was estimated in 2006 to be $3.4 billion, emphasizing the need to re-conceptualize discharge options for these patients [10].

Alternative payment models (APMs) have emerged such as Bundled Payments for Care Improvement (BPCI) and Comprehensive Care for Joint Replacement (CJR) [9, 16]. These APMs hold hospitals responsible for each patient’s 90-day episode of care, beginning on the day of hospital admission. The APM model encourages hospitals, physicians, and post–acute care providers to work together to improve care quality and coordination, from initial hospitalization through recovery, while limiting the overuse of services. This represents a shift in health care toward value-based care (with value defined as quality divided by cost), creating opportunities for innovation in the use of technology.

The emphasis on value-based care also involves a shift in discharge patterns to lower care costs. This shift has been supported by studies that have demonstrated that as discharge home with in-home PT has become more prevalent, readmission rates have not increased and outcomes have not suffered, thereby swinging the pendulum in favor of discharge home with PT in the home after total hip or knee arthroplasty [4, 7, 16, 17]. Home PT offers a transition of care, providing clinical supervision to assist in recovery in the time between hospital discharge and the start of outpatient PT. The immediate post-discharge period is crucial; 60 to 70% of all readmissions for arthroplasty patients occur within 2 weeks of hospital discharge [25]. Home-based PT allows therapists to direct patients during this time in following discharge instructions, with the goals of advancing recovery and preventing adverse events.

Few options exist, however, for patients who require supervision but do not need formal home-based PT before beginning outpatient PT. At Hospital for Special Surgery (HSS), Medicare CJR data for four quarters (from the third quarter of 2016 to the second quarter of 2017) shows that patients who were discharged home with no services had higher readmission rates than did those who were discharged home with home-based PT or to extended care facilities. As a result, home-based PT is often prescribed anyway, despite Risk Assessment and Prediction Tool (RAPT) score recommendations, to mitigate risk of re-admission and to ensure patients are supervised upon discharge. This potential overuse of home care services leads to unnecessary spending for patients who require some oversight but not the full extent that traditional home-based PT provides.

Monitoring patients in their homes with telehealth technology offers a discharge option that can minimize such overuse of services while providing needed care for patients who do not require home PT but do require monitoring and guidance. Telehealth may be a way of limiting costs in a bundled care arrangement, while also improving communication with the hospital surgical team throughout the episode of care and reducing risks to patients. Several studies that have examined the efficacy of telehealth rehabilitation after TJA involved the use of additional technology, were performed in a simulated home environment, or were performed after inpatient rehabilitation [3, 12, 13, 19, 23]. While these studies demonstrated favorable outcomes including cost savings, comparable effectiveness to conventional care for total knee arthroplasty patients, and patient satisfaction, they did not explore telehealth that makes use of existing technology in patients’ homes or for patients in bundled care programs [8, 12, 13, 19, 23]. Moreover, none were described as being integrated into the hospital electronic medical record (EMR), and many were performed outside the USA, where payment models and costs of care differ.

The purpose of this case series was to describe the effects of a pilot implementation of a physical therapist–led and hospital-EMR-integrated post-acute telehealth care navigation program. Patients who participated in the program, called HSS@Home, in lieu of traditional home-based PT were enrolled in Medicare’s CJR bundled payment arrangement. HSS@Home made use of existing technology in patients’ homes to communicate with the clinical team.

## Patients and Methods

This retrospective case series was approved by the institutional review board at HSS and conducted from July 1, 2018, to January 3, 2019. Pre-operatively, each CJR patient received a call from a case manager, who performed the RAPT. The RAPT is a pre-operative survey designed to predict discharge disposition after TJA and has been validated in total hip and total knee arthroplasty populations [5, 21]. The RAPT consists of a series of demographic questions, including age, gender, community support, and functional mobility prior to surgery, generating a score ranging from 0 to 12. A patient with a score of less than 6 will likely need to be admitted to an inpatient rehabilitation program, of 6 to 9 can likely be discharged home with additional intervention, and of more than 9 can likely go home without additional services. Inclusion criteria for the program were patients who scored 6 or greater on the RAPT; lived in the five boroughs of New York City or Westchester or Nassau Counties; had a smartphone, computer, or tablet with Wi-Fi; and felt comfortable using telehealth technology. Patient referrals were emailed to the HSS@Home program from the social work case manager. Exclusion criteria were patients who were not CJR; lived in any state other than New York; had no smartphone or home computer with camera and microphone, internet, or mobile network access; or were discharged with staples or on warfarin therapy.

The program was led by a PT navigator, a physical therapist with over 10 years of experience working with orthopedic surgery patients. The PT navigator called referred patients pre-operatively to discuss the program and review which apps to download (MyChart, powered by Epic, and Zoom Cloud Meetings). MyChart allowed patients to access their hospital-based patient portal where all telehealth appointments were visible. The Zoom app was the conduit for a Health Insurance Portability and Accountability Act (HIPPA)–compliant video link that was integrated into our EMR. Once the patient downloaded the apps, a test telehealth visit was performed to confirm the technology worked for the patient and to provide information on the telehealth program.

At HSS, as standard care, the patient’s discharge plan is reviewed post-operatively by an interdisciplinary team of nurses, case managers, physician assistants, physicians, nutritionists, and physical therapists. Patients in our program were also assessed post-operatively to confirm appropriateness for telehealth monitoring. When confirmed by the team, the patient met in the hospital with the PT navigator to establish rapport and review how telehealth visits would proceed after discharge. The first telehealth appointment occurred within 24 h of hospital discharge, and each patient received approximately two to three visits a week for 3 to 4 weeks.

Telehealth visits consisted of a home safety evaluation, answers to general health questions, review of home exercise program, and assessment of current pain level, ability to ambulate, and functional mobility. The video component allowed the PT navigator to observe the patient’s gait pattern and quality of movement to better assess patient status and to provide appropriate intervention. The PT navigator answered patients’ questions on safety and guided them on increasing exercise repetitions and ambulation distance.

When medical issues arose that were outside the scope of the PT navigator, she queried either the post-operative nurse practitioner group, which was conveniently situated so as to work cohesively to address all aspects of patient care, or the surgical team. Communication between the PT navigator and post-operative nurse practitioners, surgeon, and other staff ensured the safety and progression of telehealth patients, as some situations required immediate intervention. As patients progressed, the PT navigator assisted with selecting clinics for outpatient PT appointments. Patients were discharged from the telehealth program after starting outpatient PT and/or following up with the surgeon post-operatively.

A total of 32 patients were referred to HSS@Home. Thirteen patients did not participate in the telehealth program for one or more of the following reasons: lack of comfort with technology (*n =* 3); preference for live PT in the home (*n =* 6); not progressing as expected post-operatively and the team determined that in-home PT was most appropriate (*n =* 3); or outside New York (*n =* 1) (we were unable to accept Connecticut or New Jersey patients until PT licensure was completed for those states). Nineteen patients completed the telehealth program during its 5-month pilot period. Sixteen of the 19 patients used their smartphone for technological communication, one patient used a desktop computer, one patient used an iPad, and 1 patient used a combination of smartphone and iPad. The nine men and ten women were an average of 69 years old (range, 65 to 78 years, excluding one outlier at age 40) and scored an average of 10 on the RAPT. All patients were seen within 24 h of hospital discharge and on average for 3 weeks, nearly 3 sessions per week, for an average total of 11 visits. Participants had undergone total knee or total hip arthroplasty, including anterior, posterior, and modified precaution posterior approaches (Table 1).

**Table 1:** Patients participating in HSS@Home

Patient	Procedure	RAPT score	Age	Gender	Hospital length of stay (h)	Total days on telehealth service	Physical therapy treatment	Number of outreaches to medical team^a^	Reasons for outreach to medical team^a^	Patient feedback^b^
1	THA Anterior	11	67	Male	34	33	Home safety Ambulation Therapeutic exercise Assistive device progression Patient education	1	Possible stitch abscess. Came back to HSS to have treated.	“Great, really helpful to have someone to reach out to.”
2	THA Modified	11	66	Female	31	36	Home safety Ambulation Therapeutic exercise Patient education	3	Note for work. Prescription for outpatient PT. Review of instructions to hold dental procedures for 3 months.	“Nothing. I would change about the program. It was very helpful.”
3	THA Anterior	11	75	Male	29	25	Home safety Ambulation Therapeutic exercise Assistive device progression Transfers Patient education	1	Patient with dizziness with positional changes. Patient had blood pressure cuff at home and instructed to take blood pressure supine, sitting, and standing. Readings sent to team and monitored for several days, Symptoms resolved.	“It was great. Very professional. With the outside companies, you never know who you are going to get. This is what HSS does and it was great to get the information to the MD so quickly. Would highly recommend”
4	TKA	10	68	Female	31	18	Home safety Ambulation Therapeutic exercise Assistive device progression Patient education	2	Patient reporting increased pain, no trauma. Bleeding after dressing removal; resolved.	Sent letter: “There is no question that this service facilitated my recovery.”
5	TKA	11	69	Male	27	30	Home safety Ambulation Therapeutic exercise Patient education	3	Incision check. Check in with surgeon and nurse practitioner aftercomplaints of pain from a hamstring pull.	Sent letter: “Thank you for your new program. I hope every patient gets to experience it.”
6	THA Posterolateral	10	70	Female	48	40	Home safety Ambulation Therapeutic exercise Assistive device progression Patient education	2	Incision check. Review of removal of dressing.	“This new form of PT is so easy, manageable and comfortable.”
7	THA Posterolateral	10	68	Female	34	21	Home safety Ambulation Therapeutic exercise Patient education	2	Prescription for outpatient PT. Incision check.	“Would give it 5 stars and have told my friends who are set for surgery to go with this program.”
8	THA Modified	Missing	65	Male	33	32	Home safety Ambulation Therapeutic exercise Assistive device progression Patient education	2	Patient stopped taking blood thinner with a few days left. Verify surgeon’s post-operative protocol.	“Great, very helpful.”
9	THA Posterolateral	10	71	Female	33	19	Home safety Ambulation Therapeutic exercise Assistive device progression Patient education	2	Concern regarding diarrhea. Incision check.	“Really helpful, great.”
10	TKA	9	77	Female	48	9	Home safety Ambulation Therapeutic exercise Assistive device progression Patient education	3	Prescription for outpatient PT. Incision checks: blister near incision.	“Great, never felt alone in this process. Always had someone to reach out to.”
11	THA Anterior	10	70	Female	32	20	Home safety Ambulation Therapeutic exercise Assistive device progression Patient education	1	Prescription for outpatient PT.	“It was good, worked really well for me.”
12	TKA	10	67	Female	48	26	Home safety Ambulation Therapeutic exercise Assistive device progression Transfers Patient education	2	Prescription for outpatient PT. Concern regarding bruising.	“It was great, cannot say enough good things about it!”
13	THA Anterior	11	69	Male	29	10	Home safety Ambulation Therapeutic exercise Assistive device progression Patient education	1	Clarified duration of cane use with surgeon.	“Good, quick, easy to manage.”
14	THA Posterolateral	9	78	Female	14	17	Home safety Ambulation Therapeutic exercise Assistive device progression Patient education	3	Patient reporting 15/10 pain day after discharge. Wound check. Confirmation with surgeon whether bathing allowed.	Sent letter in gratitude for program.
15	THA Posterolateral	10	68	Male	27	29	Home safety Ambulation Therapeutic exercise Assistive device progression Patient education	1	Prescription for outpatient PT.	“Great, so helpful!”
16	THA Posterolateral	11	66	Male	27	21	Home safety Ambulation Therapeutic exercise Patient education	3	Multiple complaints or chills, overall malaise. Patient followed up with primary carephysician; no issues noted.	“PT was great and easy to do.”
17	THA Posterolateral	10	40	Male	39	24	Home safety Ambulation Therapeutic exercise Assistive device progression Patient education	1	Prescription for outpatient PT.	“Really great to stay connected to HSS.”
18	THA Posterolateral	Missing	69	Female	15	27	Home safety Ambulation Therapeutic exercise Assistive device progression Patient education	2	Prescription for outpatient PT. Question regarding supplement.	“Communication back to the surgeon is so helpful. Always felt like someone was there.”
19	THA modified	Missing	65	Male	32	20	Home safety Ambulation Therapeutic exercise Assistive device progression Patient education	1	Prescription for outpatient PT.	“Great, unsure about it to start with, but really worked very well. Would do it again.”

*THA,* total hip arthroplasty, *TKA* total knee arthroplasty, *RAPT* Risk Assessment and Prediction Tool, *PT* physical therapy

^a^Medical team: surgeon, physician assistant, licensed nurse practitioner, registered nurse

^b^Comments directly from patients at completion of program

## Results

None of the 19 patients enrolled in HSS@Home were readmitted to the hospital while participating in the telehealth rehabilitation program. The nurse practitioner group was consulted for all 19 patients and for predominantly non-emergent reasons, including prescriptions, wound checks, and medication adjustments. Other scenarios that required intervention were handled through the program. These included an incision which resulted in the patient returning to the hospital for treatment of a stitch abscess. Real-time feedback and virtual treatment was performed when a dressing was removed and an incision was still bleeding. In addition, one patient experienced low blood pressure and another experienced continued fatigue and malaise post-operatively. Patients reported satisfaction with HSS@Home, with all providing positive feedback directly to the PT navigator and four sending written letters of appreciation to hospital administration for creating this additional PT option.

## Discussion

As health care continues to change, so does the way clinical care is provided to patients. Telehealth can be used to bridge the care needs of a specific subset of patients who would benefit from continued intervention but do not require home PT services. This case series demonstrated that all 19 participants who had undergone total hip or knee arthroplasty were successfully guided in home exercise and ambulation training, prior to beginning outpatient PT, in our PT-led, EMR-integrated, telehealth navigation program in lieu of traditional home PT. Patients’ feedback to clinicians was that they were highly satisfied. Physicians reported satisfaction, as well, and requested that the program be expanded to include patients of all insurance payers.

The limitations of this case series include its small sample size, its disproportionate number of total hip arthroplasty patients (*n =* 15) vs total knee arthroplasty patients (*n =* 4), its retrospective and descriptive design, and its lack of validated measures used to assess patient satisfaction and functional performance. It may also have been affected by the biases inherent in case series, such as selection bias, limiting the generalizability of our findings to broader patient populations. We also cannot generalize our findings to patients with a 6-to-9 RAPT score because our participants scored 9 or greater. Despite these limitations, we believe our findings provide compelling preliminary data that may prove useful to future researchers in investigating the applicability of telerehabilitation technologies to patients after TJA.

“Telehealth” is a term used by several entities and defined in a variety of ways (Table 2). Yet despite the numerous definitions of “telehealth,” “telemedicine,” and “telerehabilitation,” commonalities include the use of technology to communicate and provide clinical care to a patient, who is not present in person, with the goal of improving health outcomes.

**Table 2:** Definitions of telehealth

Organization	Telehealth definition
Health Resources & Services Administration (HRSA)	The use of electronic information and telecommunications technologies to support long-distance clinical health care, patient and professional health-related education, public health and health administration. Technologies include videoconferencing, the internet, store-and-forward imaging, streaming media, and terrestrial and wireless communications [6].
World Health Organization (WHO)	The delivery of health care services, where distance is a critical factor, by all health care professionals using information and communication technologies for the exchange of valid information for diagnosis, treatment and prevention of disease and injuries, research and evaluation, and for the continuing education of health care providers, all in the interests of advancing the health of individuals and their communities [24].
American Telemedicine Association (ATA)	The remote delivery of health care services and clinical information using telecommunications technology. This includes a wide array of clinical services using internet, wireless, satellite, and telephone media [2].
Centers for Medicare & Medicaid Services (CMS)	The use of telecommunications and information technology to provide access to health assessment, diagnosis, intervention, consultation, supervision, and information across distance. Telehealth includes such technologies as telephones, facsimile machines, electronic mail systems, and remote patient monitoring devices which are used to collect and transmit data for monitoring and interpretation [11].
American Physical Therapy Association (APTA)	The use of electronic communication to remotely provide health care information and services [1].

Numerous studies demonstrate the value of telehealth with regard to cost savings, effectiveness comparable with face-to-face care for total knee arthroplasty patients, and patient satisfaction [8, 12, 13, 19, 20, 23]. Ponnusamy et al. suggested that in-home telerehabilitation and outpatient rehabilitation may provide equivalent or better outcomes compared with skilled nursing facilities [17]. A majority of the research regarding telerehabilitation and arthroplasty has been focused on total knee arthroplasty [14]. In a systemic review, Pastora-Bernal et al. suggest that telerehabilitation is a practical alternative to face-to-face rehabilitation for patients after total knee arthroplasty and that strong evidence supports the use of telerehabilitation for patients undergoing total hip arthroplasty [15].

It is difficult to compare the findings of this case series with those of other studies using telehealth due to the specific nature of HSS@Home: we treated only patients who were part of Medicare’s CJR bundled payment program, in an urban US setting, without the use of additional devices, and integrated into the hospital’s EMR. The studies we reviewed either did not specify whether patients were part of a bundled care arrangement or were conducted outside of the USA, where payment models differ [3, 8, 12–14, 19, 23]. Payment model and region may inform discharge tendencies. Ponnusamy et al. demonstrated that patients in the Northeast were more likely to be discharged to extended care facilities or inpatient rehabilitation than patients in other regions [17].

HSS@Home used patients’ own devices and technology to ease their active participation. This differs from other studies that have used additional technology, such as iPads, a telehealth video conferencing system, and a Food and Drug Administration–approved virtual outpatient physical therapy platform [8, 12, 14, 18, 23]. We decided to use existing technology for several reasons. First, the program aimed to avoid additional costs of hardware, delivery, set up, and return of items, maintenance, and damaged or lost devices. Second, using external devices could prevent last-minute additions to the program; a patient might not be able to wait for equipment delivery and set up, and pre-operative delivery would be wasteful if a patient needed a change in discharge plan. Moreover, patients may be more comfortable with using their own equipment. Overall, using patients’ existing technology allowed for flexibility, cost savings, and ease of use.

Several studies describe patient satisfaction with telehealth programs [8, 13, 19, 22]. Moffet et al. concluded that patient satisfaction for telehealth was comparable with face-to-face intervention for TKA patients [13]. Russell et al. reported an average patient satisfaction score of greater than 9 on a 10-cm visual analog scale (with the exception of a question on the visual quality of videoconferencing) and indicated they would use telehealth again and recommend it to friends [19]. Kairy et al. reported that all participants agreed that telehealth rehabilitation treatment was a good alternative to in-person care [8]. While we used no formal patient satisfaction measures in our case series, each patient provided positive feedback about their experiences, with four writing formal letters of appreciation to hospital leadership regarding HSS@Home (Table 1). Feedback from both patients and physicians confirmed that HSS@Home added value and was worthy of further exploration and expansion.

Our use of an experienced hospital-based physical therapist to lead this program ensured that the desired post-operative guidelines and surgeon preferences were followed. When medical issues arose that were outside the scope of PT practice, the post-operative nurse practitioner group was easily accessed and consulted. Communication to the surgical team was further enhanced by the telehealth program being integrated into the hospital’s EMR and patient portal. The visit schedule and clinical documentation were easily visible to the surgeon and interdisciplinary team, allowing for easy tracking. A single sign-on into the portal allowed patients to see their upcoming visits, communicate with the medical team, and access discharge information. None of the studies we reviewed referred to this type of interdisciplinary arrangement, and we could not ascertain whether other studies used the EMR in a similar way.

There are several directions for the growth of HSS@Home and future research. First, we suggest the use of physical performance and patient-reported outcomes at designated time points to track effectiveness, satisfaction, and long-term outcomes. Second, we suggest expanding the program to include patients covered by commercial payers. Third, we suggest implementing a patient-facing digital platform for delivering additional content, enabling advancement of home exercises and ambulation. This platform could also track compliance with home exercises and overall progress with recovery, wherein patient input would trigger appropriate interventions to be provided. Fourth, we suggest extending the duration of HSS@Home to cover up to a year post-surgery, to facilitate continued compliance with surgeon-specific protocols and provide opportunities for long-term follow-up. Future research should include a comparative, prospective design with sample size determined by power analysis.

In conclusion, HSS@Home proved to be a promising alternative to home-based PT that was effective in the monitoring of 19 patients after hip or knee arthroplasty, resulting in no readmissions and avoiding overuse of home care. This case series sets the stage for future research in the use of telehealth technology in providing rehabilitative care to TJA patients.

## Electronic supplementary material

ESM 1 (PDF 1224 kb)

ESM 2 (PDF 1224 kb)

ESM 3 (PDF 1224 kb)

ESM 4 (PDF 1224 kb)

ESM 5 (PDF 1224 kb)
